# Comparative Study of Carcass Characteristics and Meat Quality of Local Mediterranean Donkey Breeds

**DOI:** 10.3390/foods13060942

**Published:** 2024-03-20

**Authors:** Mohamed Aroua, Hayet Haj Koubaier, Chaima Rekik, Antonella Fatica, Samia Ben Said, Atef Malek, Mokhtar Mahouachi, Elisabetta Salimei

**Affiliations:** 1 Université de Jendouba, Ecole Supérieure d’Agriculture du Kef, LR: Appui à la Durabilité des Systèmes de Production Agricoles du Nord-Ouest, Complexe Universitaire Boulifa, Le Kef 7119, Tunisia; chaima.rekik2020@gmail.com (C.R.); sabensaid@gmail.com (S.B.S.); taymallahmah@gmail.com (M.M.); 2 Laboratory of Innovation and Valorization for Sustainable Food Industries, High School of Food Industries of Tunis, University of Carthage, El Khadra 1003, Tunisia; h.kbaier@gmail.com; 3 Dipartimento Agricoltura, Ambiente e Alimenti, Università Degli Studi del Molise, Via de Sanctis SNC, 86100 Campobasso, Italy; antonella.fatica@unimol.it; 4 Animal Nutrition Laboratory, National School of Veterinary Medicine Sidi Thabet, University of Mannouba, Ariana 2020, Tunisia; malek.atef@iresa.agrinet.tn

**Keywords:** meat quality, donkey, amino acids, fatty acids

## Abstract

This study aimed to evaluate carcass and meat quality traits in Masri (*n* = 14) and North African (*n* = 14) male donkeys, raised in a semi-intensive breeding system, grazing on mountainous areas, with supplementation of 1 kg of barley per day per animal, slaughtered at 5 (±0.48) years old. Compared to Masri, the North African population exhibited higher (*p* < 0.05) final body weights (181.7 ± 2.1 and 212.5 ± 7.6 kg) and cold carcass weights (101.7 ± 1.3 and 116.2 ± 4.5 kg), but lower (*p* < 0.05) cold dressing percentages (56.0 ± 0.4 and 54.6 ± 0.4%). Meat quality analyses showed higher (*p* < 0.05) cooking loss values in Masri meat (43.9 ± 0.8 vs. 39.9 ± 1.2%). Among the meat color parameters, the hue value was higher (*p* < 0.05) in North African samples (0.42 ± 0.01 vs. 0.39 ± 0.1). Meat from North African donkeys had higher (*p* < 0.05) dry matter, fat, and protein contents. Meat amino acid analysis revealed abundant levels of lysine, leucine, and methionine, in both populations. Donkey meat from both populations presented a high polyunsaturated fatty acids content, resulting in polyunsaturated fatty acids/saturated fatty acids and omega 6/omega 3 ratios for all breeds close to the recommended values for human health. Atherogenic and thrombogenic indices were also suggested to have positive effects on consumers’ artery health. The characteristics of donkey meat present intriguing nutritional aspects compared to ruminant meat, and its production should be encouraged in the rural development of inner-Mediterranean areas.

## 1. Introduction

The world donkey population is estimated to be over 40 million individuals consisting of 185 recognized breeds [1]. Donkeys have a long history of being used for pack and draft purposes. They have been the preferred mode of transportation in mountainous regions since their domestication in the Middle East and Egypt around the 3rd millennium BCE [2,3]. Despite the advancement of mechanization in agriculture, donkeys remain extensively utilized in Africa and Asia [4,5].

In recent times, there has been a renewed worldwide interest in donkeys due to the exceptional quality of their milk and meat. Donkey meat is considered a sustainable source of high-quality and eco-friendly meat [6,7], with excellent nutritional value owing to their access to large pastures in the equine breeding system [8,9].

Donkey meat presents appealing quality characteristics, including low fat contents ranging from 1.6% to 3.5% for breeds such as Martina Franca, Amiata, Littoral Dinaric, and Istrian [10,11]. The cholesterol content varies between 55 to 68.7 mg per 100 g of meat from African and Italian donkey breeds; moreover, it is also a valuable source of iron [6,10,11,12,13]. Donkey meat exhibits noteworthy protein content, ranging from 21.8% to 23.6% for breeds like Martina Franca, Amiata crossbred, and Littoral Dinaric, respectively [10,14]. This is complemented by a favorable ratio of unsaturated fatty acids (PUFA) to saturated fatty acids (SFA) and an intriguing dietary n-6/n-3 ratio [6,13,15]. Additionally, donkey meat contains a significant amount of essential amino acids, underscoring its nutritional value when compared to other types of ruminant meat, as reported by [16].

Previous studies have demonstrated that donkey is characterized by leaner meat, with essential amino acids, and unsaturated fatty acids, including hexanal, a grass fragrance derived mainly from the oxidative decomposition of unsaturated fatty acids [10,11]. Notably, it contains less total fat and cholesterol, and its energy content is lower compared to pork, beef, and lamb meat [11]. Furthermore, the muscle fiber in donkey meat is found to be more tender than that of other domesticated animal meats [17]. The quality of donkey meat, encompassing the cooking rate, water-holding capacity, pH, color, textural parameters, and contents of protein, hydroxyproline, fat, and lactate, is influenced by various factors, including the animal breed, farm management, and animal nutrition [18].

China leads in donkey meat production followed by Niger and Burkina Faso [1]. Additionally, equine meat consumption is traditional in several European countries such Italy, Croatia, and France [10]. In Tunisia, the annual production of donkey meat stands at 4000 tons [19]. Despite a substantial population of approximately 123,000 autochthonous donkeys [8], there is a noticeable dearth of research focusing on these indigenous breeds. The utilization of indigenous donkey breeds like Masri and North African for meat production holds potential social and economic significance, as it contributes to the augmentation of local production systems. This practice not only adds value but also plays a crucial role in promoting and safeguarding animal biodiversity. Such an approach further enhances local production systems by capitalizing on the inherent adaptability and disease resistance observed in donkeys, as highlighted by [20].

This study aims to comprehensively evaluate the carcass characteristics and meat quality of the Masri and North African donkey populations. By shedding light on these aspects, the research aims to provide crucial insights into the untapped potential of these donkey breeds for meat production, while also contributing to the broader goal of biodiversity preservation.

## 2. Materials and Methods

### 2.1. Informed Consent

The Review Board of the Laboratoire D’appui à la Durabilité des Systèmes de Production Agricoles du Nord-Ouest, Ecole Supérieure d’Agriculture du Kef, Université de Jendouba Boulifa 7119, Kef, Tunisia, approved the research protocol of the present study. Both the slaughterhouse company “Ellouhoum”, Tunis, Tunisia, and the director of the Tunisian Society for protection of animals abroad provided written consent.

### 2.2. Animals and Meat Sampling

A total of 28 male donkeys, on average 5 (±0.48) years old, were enrolled in the study, with 14 subjects belonging to the Masri population and 14 to the North African population. Breeds do not differ in adult weight. All donkeys, raised in the same breeding system, as previously described [8], were kept on grass pasture in a mountainous area, with free access to water and daily supplementation with barley (1 kg/head) to match the animals’ nutritional needs. For the present study, donkeys were transported to Ellouhoum Company, a slaughterhouse under the supervision of the Tunisian Ministry of Trade and Export Development. Animals were slaughtered following a halal procedure, carried out by specialized personnel who adhered strictly to good animal husbandry practices as established by national authorities (Law No. 2005-95, 18 October 2005). The halal slaughter procedure ensures the welfare of the animals and follows ethical principal guidelines set by the Institution de la Recherche et de l’Enseignement Supérieur Agricole of Tunisia.

In the slaughterhouse, donkeys’ live weights were recorded, and carcasses were weighed and transferred to a cold room, maintained at a temperature of 4 °C, for storage. After 24 h, the weights of the cold carcasses were recorded, and the cold dressing percentage was calculated. Muscle samples of the *Longissimus thoracis* (LT) were collected from the right side of each carcass, specifically between the 9th and 13th ribs, 24 h post-slaughter. The average weight of each muscle sample was 400 g per animal and was carefully placed in vacuum-sealed bags to preserve their freshness and then stored at 4 °C. Subsequently, the samples were transferred to the laboratory for further analyses and examination of the present study.

### 2.3. Physicochemical and Chemical Analyses

The pH measurements were assessed 24 h post-mortem after muscle excision. For this, 10 g of meat samples were homogenized with 90 mL of distilled water using a blender (Moulinex, Groupe SEB, 21120, Site d’Is-sur-Tille, Dijon, France); for about 1 min after that, the pH was recorded [21]. The pH values were measured using a calibrated digital pH meter (model-HI 98107 pHep HANNA Instruments, Carrollton, TX, USA) after homogenization.

Forty-eight hours post-slaughter, 50 g of donkey meat was extracted from each LT sample for chemical composition analysis. Simultaneously, the water content in 5 g minced meat samples underwent drying in a 105 °C oven, following the AOAC 950.46 standard [22]. The total protein content was determined by the dosage of the total nitrogen (N) of the meat sample according to the Kjeldahl method (AOAC 928.08, [22]). The principle of this method consists of a digestion of approximately 1 g of a sample by heating in the presence of concentrated sulfuric acid and a catalyst. After alkalization of the reaction products, the released ammonia is distilled and trapped in a solution of boric acid. Then, it is titrated with a solution of hydrochloric acid. Once the total nitrogen is determined, it is converted to a protein content (%) using the following formula: protein content (%) = N (%) × 6.25. The ash content was evaluated through mineralization at 550 °C per AOAC 920.153 [21]. The intramuscular fat content was determined following the Soxhlet method (AOAC method 991.36) [22]. First, the meat sample is treated with boiling diluted hydrochloric acid to release the included and bound lipid fractions. After filtration, the resulting mass was dried, placed in a porous thimble, and extracted in petroleum ether until the solute was completely exhausted in the sample. After completion of the extraction, the solvent was evaporated and the mass of the lipid was measured.

The assessment covered moisture [23], protein [24], fat [25], and ash [26] contents, utilizing methods also acknowledged by the International Organization for Standardization (ISO).

The determination of myoglobin [27] was executed as follows: from each animal a portion of the LT muscle sample was finely ground using a meat-grinder machine (Talsa w 98 L, 46950 Xirivella, Valencia, Spain). A ground meat sample of 5 g was weighed and placed in an identified glass bottle. To this, 1 mL of distilled water and 20 mL of acetone were added successively. The contents were thoroughly mixed to ensure homogeneity, and then 0.5 mL of hydrochloric acid (12 N) was added. The vials were tightly closed, agitated, and stowed away in a dark, enclosed area for 24 h. The next day, after filtration, the optical density of the filtrate was carried out using a spectrophotometer at a wavelength of 513 nm (s6305, Jenway, Dunmow, Essex, UK). The myoglobin content was calculated as follows:

(1) $$Myoglobin\left(\frac{mg}{g}fresh\;muscle\right)=Absorbance\times 8.816$$

### 2.4. Color Parameters Measurement

The color of donkey meat muscle samples collected from LT, between the 10th and 11th ribs, was evaluated according to [13], 48 h after the animals’ slaughter and muscle excision using a Chroma Meter (model CR-400, Konica Minolta Holdings, Osaka, Japan) with a measured area of 8 mm and a 10° view angle, and a D65 illuminant was used to evaluate color. The results were expressed as the average of three measurements conducted on a cut surface area after 30 min of blooming (with LT exposed in air) at room temperature (20 °C), utilizing the CIE system. Chroma (C*) and hue (h*) values were calculated based on observed data on lightness (L*), redness (a*), and yellowness (b*): h* = arctangent(b*/a*); C* = √(a^2^ + b^2^). The measuring lens, when placed on the meat surface, was rotated through 0°, 45°, and 90° (clockwise) to obtain three distinct reflectance measurements which were subsequently averaged.

### 2.5. Determination of Total Amino Acid Profile

Freeze-dried samples of meat using Labconco lyophilizer (freezone1, Kansas City, MO, USA) were hydrolyzed using 6 M hydrochloric acid (HCl) for 22 h at a temperature of 110 °C. Cysteine and methionine were oxidized to cysteic acid and methionine sulphone, respectively, prior to hydrolysis. The liquid phase (hydrolysate) was further processed by filtration through a 0.45 μm filter to remove any remaining solid particles.

The resulting filtered supernatants were then used for the amino acid analysis. To determine the amino acid profiles, a high-performance liquid chromatography (HPLC) system was used (Biochrom 30 series AA analyzer, Biochrom Ltd., Cambridge Science Park, UK) equipped with a resin column exchange (20 × 0.46 cm inner diameter). Peaks were matched and identified by comparing their retention times with those of standard amino acids. In the determination of tryptophan content, a two-step process was implemented. Initially, alkaline hydrolysis was conducted using lithium hydroxide (LiOH) at a temperature of 110 °C for a duration of 16 h. Subsequently, the resulting hydrolysates underwent a spectrometric analysis at 590 nm using 4-dimethylaminobenzaldehyde (DMAB).

### 2.6. Analysis of Fatty Acid Composition

The fat content of the meat was extracted using the chloroform–methanol extraction technique reported in the literature [28]. The fatty acid analysis was performed using methyl esters produced using direct trans-esterification according to the ISO 12966-2:2017 standards [29]. The fatty acid composition of samples was analyzed using a capillary gas chromatograph (AGILENT 6890 N, Santa Clara, CA, USA) equipped with a flame-ionization detector and an HP-88 fused-silica capillary column (100 m × 0.25 mm × 0.2 µm film thickness). The separation of fatty acids was conducted using the following temperature program: the initial temperature was set at 100 °C for 3 min, and then it was increased to 240 °C at a rate of 5 °C per minute and held for 10 min at the final temperature. The carrier gas used was hydrogen with a flow rate of 1 mL/min. The detector temperature was set at 260 °C, and the injector temperature was set at 255 °C with a splitting ratio of 50%. Fatty acid peaks were identified by comparing their retention times with those of standard fatty acids.

The atherogenic index (AI) and thrombogenic index (TI) were calculated based on the method described by [30] as follows:

(2) Atherogenic Index (AI)=(C12:0+(4×C14:0+C16:0))/(MUFA+PUFA)

(3) Thrombogenic Index (TI)=(C14:0+C16:0+C18:0)/[(0.5×MUFA)+(0.5×PUFA n-6)+(3×PUFA n-3)+(PUFA n-3/PUFA n-6)]

### 2.7. Determination of Cooking Loss and Water-Holding Capacity

The cooking loss (CL) was determined according to Bowker et al. [31]. To assess water cooking loss, uniform meat samples collected from the 11th to 12th rib were initially weighed (Wi: initial weight) and placed in plastic bags. Subsequently, the samples were submerged in a water bath at 75 °C and heated for 30 min until reaching an internal temperature of 75 °C, as monitored by a thermocouple [32]. Following the cooking process, the bags were cooled under running tap water and dried with paper towels. The cooked meat was then reweighed (Wf: final weight). CL was determined by calculating the percentage difference between the sample’s initial and final weights using the formula:

(4) %CL=100×(Wi−Wf)/Wi

The water-holding capacity (WHC) was assessed according to literature [31]. Briefly, a 10 g sample of minced meat was mixed with 15 mL of 0.6 M NaCl for 2 min. Subsequently, the mixture was refrigerated at 4 °C for 15 min. Afterward, the mixture was shaken and then centrifuged at 7669× *g* for 15 min. The WHC was calculated using the following formula:

(5) %WHC=[(amount of 0.6 M NaCl added−supernatant volume)/sample weight]×100

### 2.8. Statistical Analysis

No significant covariant effect of age was found in preliminary ANOVA processing of data so that a one-way analysis of variance was used to examine the disparities in meat chemicals, cooking loss, water-holding capacity, amino acids, and fatty acid composition between the two Tunisian donkey populations. To further scrutinize these distinctions, Student’s *t*-test was applied, and the findings were depicted in the form of least squares mean values, alongside their corresponding standard errors. The statistical significance threshold was set at *p* < 0.05 using the XL STAT software (2016 version, Addinsoft, Long Island, NY, USA) [33].

## 3. Results and Discussion

### 3.1. Carcass Characteristics

The data in Table 1 present a comparative analysis of body weight, cold carcass weight, and cold dressing percentage between North African and Masri donkey populations.

Compared to Masri subjects, the North African donkeys display significantly higher body weights and cold carcass weights (Table 1), whereas they show lower values of cold dressing percentages. Interestingly, the cold dressing percentages of Tunisian donkeys are within the range of values reported for Mediterranean donkeys, such as the Martina Franca breed [13,34]. These findings are consistent with values reported by Aganga et al. [35] for donkeys, which showed that carcass yield varies between 54.5% and 59.5%.

Polidori et al. [13] reported that the dressing percentage is influenced by various factors, including the stage of maturity, degree of finishing, breed, and intestinal content. As donkeys were raised in the same breeding system and marginal area, the observed performances are likely due to the breed.

### 3.2. Meat Quality

The qualitative characteristics of Masri and North African donkey meat are presented in Table 2.

Meat from the North African donkey population displays significantly higher contents of dry matter, fat, and protein when compared to values observed in the Masri donkey meat. These differences can be attributed to various factors. Firstly, morphological variations between the two populations may influence the distribution of fat and muscle tissues, leading to disparities in fat and protein content [8]. Additionally, rearing and feeding practices and behavior play a crucial role in shaping the meat’s composition [9]. In our study, the diverse feeding behavior could have contributed to the increased fat and protein levels observed in the meat. The protein content of Tunisian donkey breeds’ meat was found to be consistent with data from the Italian Martina Franca breed, which ranges from 19% to 21% [14,15]. However, it was lower than the protein content reported for Croatian breeds Istrian and Littoral Dinaric (23.56% and 23.63%, respectively) [10].

Regarding the fat content of meat, the Tunisian breeds contain from 0.98% to 4.49% of fat, which aligns with data reported for other donkey breeds [10,13,14,15].

Meat WHC did not differ between the two donkey breeds, but a significant difference was found in cooking loss, being in Masri donkey breed significantly higher than in North African (43.92 ± 0.92% vs. 39.95 ± 1.23%, respectively); at the same internal temperature averaging 74.3 (± 0.45) °C, this finding is consistent with previous studies for other donkey breeds [12]. It is worth noting that CL can be influenced by the cooking temperature [36,37].

These results suggest that the donkey meat from the North African population exhibits superior water-retention capabilities during cooking, contributing to its tenderness. Lower cooking loss is often associated with juicy and more tender meat indicating greater retention of liquid during cooking [38].

Regarding meat pH, the observed values did not exhibit significant difference between the two breeds (*p* > 0.05, Table 2). As pH is a crucial parameter that influences both the preservation and transformation of meat [39], the pH_24_ values ranged between 5.5 and 5.7. These findings were in line with previous studies [40,41]. The variation in pH between the slaughter day and 24 h after is attributed to a low level of muscle glycogen at the time of slaughter, resulting in reduced lactate production, which promotes the attainment of more tender meat. Additionally, this leads to quicker depletion of ATP and an earlier onset of rigor mortis, allowing for extended activity of proteases [42].

The high pH value of donkey meat may also indicate the presence of proteolytic processes influenced by endo- and exo-enzymes from microorganisms responsible for the maturation process [43]. However, the type of finishing diet and the rearing system are also reported to influence the pH of equine meat [9]. According to Beldarrain et al. [40], pH differences could also be linked to other factors such as physical activity and the condition of animals before slaughter, including stress levels and fasting periods.

Pre-slaughter stress related to various events that occur before the animal’s death greatly affects the occurrence of meat with higher pH levels, because of the decreased glycogen reserves in the muscles, which result in higher pH values of the meat [39].

Color plays a fundamental role in shaping consumers’ perception of meat quality and significantly affects their purchasing decisions [15,34]. It is widely regarded as an important indicator of product freshness [43]. Meat color is influenced by various factors, such as the age and sex of the animal, the rearing system, and the anatomical source of the meat cut [12].

No differences in meat color brightness (L*), redness (a*), and yellowness (b*) were observed between breeds, except for the hue (h*) parameter. The color observed exhibited a dark-brown hue with low clarity (L*), which is a typical characteristic of meat from the equid family. This observation aligns with findings reported in the literature [13,14].

The specific dark color of equine meat can be attributed to the amount of myoglobin oxygenation, which is closely related to the a* value [44,45]. Notably, equine meat tends to have a higher concentration of myoglobin in adulthood [46]. As the a* value increases and the L* value decreases, the meat appears darker [47].

The total amino acid composition of protein in Masri and North African donkey meat after HCl hydrolysis is reported in Table 3. Both the Masri and North African donkey populations exhibit insignificant (*p* > 0.05) differences in amino acid contents, providing all the essential amino acids that play a crucial role in nutrition, especially for specific population groups with distinct requirements, such as children, the elderly, and individuals with health problems. The similarity in the amino acid composition of meat from two different breeds can be attributed to various factors, including shared genetic backgrounds shaped by selective breeding practices, similar feeding regimens, and comparable environmental conditions [48].

Lysine emerges as the most abundant essential amino acid in donkey meat, aligning with prior research outcomes [6,14]. Beyond its pivotal role in protein synthesis, lysine actively engages in vital physiological functions, encompassing the generation of enzymes, hormones, and antibodies. This amino acid contributes to immune system efficacy, supports metabolic processes, and plays a crucial role in collagen formation, essential for the integrity of skin, bone, and connective tissues [49].

Following lysine, leucine claims the position of the second most abundant essential amino acid, with methionine closely following. The content of the essential amino acid tryptophan in donkey meat adheres consistently to the values documented in the existing literature [15]. Tryptophan assumes a critical role with diverse physiological implications, serving as a precursor for serotonin and melatonin—neurotransmitters that are crucial in mood regulation and sleep–wake cycles [50]. The unwavering presence of tryptophan in donkey meat, in harmony with established values, underscores the stability and reliability of this nutritional component within donkey meat.

Among the non-essential amino acids, glutamine and aspartic acid were found to be the most represented, which is in line with data reported for Italian and Croatian donkey breeds [6,9,10,13,14]. Furthermore, a considerable amount of arginine was detected. Arginine serves important functions in vascular homeostasis, spermatogenesis, and fetal growth. It is considered a conditionally essential amino acid when endogenous synthesis is insufficient to meet metabolic demands, which often occurs during the growth of children and in highly catabolic conditions [51].

Overall, Masri and North African donkey meat can be considered a rich source of essential and non-essential amino acids, i.e., proteins with a high biological value, a valuable component of a balanced and nutritious diet. As reported in Table 3, the two Mediterranean populations of donkeys exhibit elevated levels of essential amino acids compared to the overall percentage of total amino acids, as the AAE/AAT ratios over 50% show.

The results obtained for the meat fatty acid profile of the investigated Tunisian donkey populations are presented in Table 4.

The fatty acid composition of Tunisian donkey meat reveals that the two most prominent fatty acids are oleic acid (C18:1, n-9) and palmitic acid (C16:0), consistent with previous findings in meat from the Martina Franca donkey breed [14,15,16].

Tunisian donkey meat shows a balanced proportion of saturated fatty acids (SFAs) and monounsaturated fatty acids (MUFAs), accounting for approximately 38% and 34%, respectively. These results are in line with earlier studies [14]. The fatty acid profile of donkey meat can vary significantly, as it is notably influenced by the rearing system, diet, and age at slaughter [52].

The high concentration of polyunsaturated fatty acids (PUFAs) observed in North African and Masri donkey meat is a result of the leanness of this species. When the fat content is low, the contribution of phospholipids to the meat’s fatty acid profile becomes more significant. These phospholipids are more unsaturated than triglycerides. This increase in PUFAs is consistent with findings in Martina Franca donkey meat [14] and brings the PUFA/SFA and n-6/n-3 ratios in donkey meat close to the recommended benchmark values, surpassing those found in beef, lamb, and pork meat [53].

Although the atherogenic and thrombogenic indices (parameters estimating the risk of artery obstruction and atherosclerosis) are similar for both donkey populations and comparable to values from previous studies [6,14,47], it is crucial to consider the specific metabolic effects of saturated and polyunsaturated fatty acids, as fatty acids can have different impacts on the prevention or promotion of atherosclerotic and thrombotic phenomena. The atherogenic and thrombogenic nature of certain fatty acids, such as C12:0, C14:0, C16:0, and C18:0, is highlighted by these indices.

The differences in the fatty acid profile between the two studied donkey populations could be attributed to variations in phospholipid levels, which tends to be higher in red oxidative muscle fibers than in glycolytic muscle fibers [54].

It is important to note that donkey meat exhibits slightly higher values than the WHO’s recommended threshold for the n-6/n-3 ratio, mainly due to elevated levels of linoleic acid (C18:2) and arachidonic acid (C20:4) compared to ruminant meat [6].

## 4. Conclusions

The experimental results of the study comparing Masri and North African donkey breeds underscored significant differences in carcass characteristics and meat quality, positioning North African donkeys as having preferable traits such as higher final body weights, cold carcass weights, and lower cooking losses. The findings emphasize the potential value of North African donkey meat over Masri donkey meat, particularly in terms of its nutritional richness and cooking properties. Taken together, the results confirm donkey meat as a valuable source of protein and essential amino acids, in optimal quantities. Moreover, due to its high content of polyunsaturated fatty acids and a significant n-6/n-3 ratio, donkey meat can be recommended for individuals with heart problems and blood count disorders, particularly anemic individuals.

These distinctions highlight the importance of recognizing the unique qualities of donkey meat and its potential as a desirable alternative to traditional red meats in the Mediterranean area. Moreover, the study emphasized the significance of donkeys in Tunisia and advocated for the promotion of donkey breeding, indicating that with proper attention, donkeys could serve as a valuable alternative to ruminants. The potential benefits for farmers in the Mediterranean region were highlighted, suggesting that investing in donkey breeding could provide economic advantages and contribute to the sustainability of local farming practices.

## Figures and Tables

**Table 1:** Carcass characteristics of the studied breeds (means ± SE).

Traits	Donkey Breeds	
	Masri	North African
Body weight (kg)	181.7 ± 2.1 ^b^	212.5 ± 7.6 ^a^
Cold carcass weight (kg)	101.7 ± 1.3 ^b^	116.2 ± 4.5 ^a^
Cold dressing (%)	56.0 ± 0.4 ^a^	54.6 ± 0.4 ^b^

Different letters (a, b) indicate significant differences at *p* < 0.05.

**Table 2:** Chemical and physico-chemical parameters of *Longissimus thoracis* from Masri and North African donkeys.

Parameters	Breeds	
	Masri	North African
Moisture, g/100 g	76.49 ± 0.57 ^a^	72.76 ± 0.58 ^b^
Ash, g/100 g	1.09 ± 0.12	1.12 ± 0.06
Protein, g/100 g	19.52 ± 0.45 ^b^	22.11 ± 0.50 ^a^
Fat, g/100 g	0.98 ± 0.13 ^b^	4.49 ± 0.73 ^a^
Myoglobin, mg/g	8.31 ± 0.37	8.44 ± 0.24
pH_24_	5.78 ± 0.03	5.92 ± 0.07
CL (%)	43.92 ± 0.82 ^a^	39.95 ± 1.23 ^b^
WHC (%)	85.39 ± 0.60	85.64 ± 0.35
Color parameters		
L*	39.42 ± 0.43	39.79 ± 0.15
a*	15.27 ± 0.64	15.16 ± 0.39
b*	−0.94 ± 0.33	−0.55 ± 0.24
C*	17.60 ± 0.40	17.48 ± 0.20
h*	0.39 ± 0.01 ^b^	0.42 ± 0.01 ^a^

Different letters (a, b) indicate significant differences at *p* < 0.05, CL (cooking loss), WHC (water-hold capacity) L* (lightness), a*(redness), b* (yellowness), C* (chroma), and h* (hue).

**Table 3:** Amino acid composition (g/100 g) of Masri and North African donkey meat.

Amino Acid *	Masri	North African
Aspartic acid	1.67 ± 0.35	1.72 ± 0.29
Glutamine	3.14 ± 0.57	3.35 ± 0.64
Serine	0.46 ± 0.2	0.53 ± 0.24
Glycine	0.81 ± 0.3	0.79 ± 0.34
Cystine	0.19 ± 0.05	0.22 ± 0.07
Alanine	0.91 ± 0.19	0.93 ± 0.21
Arginine	1.38 ± 0.36	1.4 ± 0.38
Tyrosine	0.88 ± 0.23	0.87 ± 0.24
Proline	0.83 ± 0.26	0.84 ± 0.31
Histidine	0.82 ± 0.30	0.81 ± 0.31
Threonine	0.70 ± 0.28	0.71 ± 0.22
Valine	0.66 ± 0.18	0.72 ± 0.24
Methionine	1.09 ± 0.36	1.15 ± 0.31
Phenylalanine	0.71 ± 0.18	0.74 ± 0.17
Isoleucine	0.92 ± 0.24	0.92 ± 0.32
Tryptophan	0.23 ± 0.09	0.25 ± 0.11
Leucine	1.5 ± 0.42	1.52 ± 0.38
Lysine	2.06 ± 0.32	2.16 ± 0.41
Total (AAT)	18.96 ± 1.78	19.66 ± 2.25
Essential (AAE)	10.07 ± 1.11	10.38 ± 1.05
Ratio AAE/AAT (%)	53.11± 2.68	52.8 ± 2.82

* No significant differences (*p* > 0.05) in amino acid contents were found between Masri and Norh African donkey population.

**Table 4:** Fatty acid composition (% total fatty acids) of Masri and North African donkey meat.

Fatty Acid	Masri	North African
C11:0	0.14 ± 0.01 ^b^	0.54 ± 0.08 ^a^
C12:0	0.25 ± 0.02	0.24 ± 0.08
C14:0	2.67 ± 0.42	2.94 ± 0.54
C14:1	0.28 ± 0.08	0.36 ± 0.10
C15:0	0.47 ± 0.11	0.44 ± 0.08
C15:1	1.41 ± 0.21	1.45 ± 0.20
C16:0	24.23 ± 0.23 ^a^	23.60 ± 0.24 ^b^
C16:1	3.85 ± 0.65	4.10 ± 0.52
C17:0	0.80 ± 0.05 ^a^	0.52 ± 0.06 ^b^
C18:0	9.85 ± 0.72	8.56 ± 0.90
C18:1 n-9	26.10 ± 1.02	25.80 ± 0.82
C18:2 n-6	20.40 ± 1.4	21.50 ± 1.26
C18:3 n-3	2.99 ± 0.92	3.21 ± 0.52
C20:0	0.56 ± 0.09 ^b^	0.85 ± 0.12 ^a^
C20:1 n-6	0.34 ± 0.10	0.33 ± 0.14
C20:3 n-3	0.17 ± 0.04	0.19 ± 0.06
C20:3 n-6	0.47 ± 0.05 ^b^	0.55 ± 0.03 ^a^
C20:4 n-6	2.21 ± 0.42	2.44 ± 0.39
C20:5 n-3	0.17 ± 0.02	0.15 ± 0.03
C22:1	2.65 ± 0.56	2.24 ± 0.48
SFA	38.96 ± 1.52	37.69 ± 1.48
MUFA	34.63 ± 3.30	34.28 ± 3.32
PUFA	26.41 ± 2.61	28.04 ± 2.70
PUFA/SFA	0.76	0.81
n-3	3.33 ± 0.20	3.55 ± 0.24
n-6	23.42 ± 0.92	24.82 ± 1.32
n-6/n-3	7.03	6.99
AI	0.57	0.57
TI	0.93	0.87

Different letters (a, b) indicate significant differences at *p* < 0.05.

## Data Availability

The original contributions presented in the study are included in the article, further inquiries can be directed to the corresponding author.
